# Mathematical insights into epidemic spread: A computational and numerical perspective

**DOI:** 10.1371/journal.pone.0323975

**Published:** 2025-06-10

**Authors:** Amer Alsulami, Naveed Shahid, Nauman Ahmed, Mohammed Almalahi, Alaa Mustafa, Khaled Aldwoah

**Affiliations:** 1 Department of Mathematics, Turabah University College, Taif University, Taif 21944, Saudi Arabia; 2 Department of Mathematics and Statistics, The University of Lahore, Lahore, Pakistan; 3 Department of Mathematics, College of Computer and Information Technology, Al-Razi University, Sana’a, Yemen; 4 Jadara University Research Center, Jadara University, Irbid, Jordan; 5 Department of Mathematics, Faculty of Science, Northern Border University, Arar, Saudi Arabia; 6 Department of Mathematics, Faculty of Science, Islamic University of Madinah, Madinah 42351, Saudi Arabia; University of Dhaka, BANGLADESH

## Abstract

This study aims to investigate and analyze the dynamics of diarrhea infectious disease model. For this purpose, a classical diarrhea disease model is converted into the diffusive diarrhea epidemic model by including the diffusion terms in every compartment of the system. Basic assumptions of the proposed model are described for a vivid understanding of the model’s behavior. In addition, the pros and cons of the proposed model for short and long terms behavior of the diffusive system are presented. The system has two steady states, namely the disease-free equilibrium and endemic equilibrium points. The system is analyzed, analytically by ensuring the positivity, boundedness and local, and global stability at both the steady states. Moreover, the implicit nonstandard finite difference scheme is designed to extract the numerical solutions of the diffusive epidemic model. To ensure the reliability and efficacy of the numerical scheme, the positivity, consistency and both linear and nonlinear stabilities are presented by establishing some standard results. Simulated graphs are sketched to study the nonlinear behavior of the disease dynamics. All the graphs depict the positive, bounded and convergent behavior of the projected numerical scheme. Also, the numerical graphs reflect the role of the basic reproductive number, *R*_0_, in attaining the steady state. The article is closed by providing productive outcomes of the study.

## 1 Introduction

Diarrhea is not an epidemic, but it can be a symptom of some diseases. Depending on its underlying cause, diarrhea may contribute to epidemic breakouts, particularly when infectious organisms, such as parasites, bacteria, or viruses cause it. Symbolically, it is a medical disorder of the human body characterized by frequent passing of loose, watery stools [1]. People of all ages may be affected by this frequent gastrointestinal issue. Usually, diarrhea has two stages one is acute and the other is chronic. In the acute stage, diarrhea rests for a few days, while in the chronic stage, it persists for several weeks or longer. Diarrhea is frequently a sign of an underlying illness, but if left untreated, it can also result in electrolyte imbalances and dehydration. Numerous things, including infections, viral infections, bacterial infections, and parasite diseases, can also cause diarrhea. In viral infections, common viruses that can cause diarrhea are norovirus, rotavirus and enteric adenovirus. In bacterial infection, Campylobacter, Salmonella, Escherichia coli and Shigella are among the bacteria that commonly cause diarrhea, which is typically brought on by contaminated food or drink. For parasitic infections, *Entamoeba histolytica* and *Giardia lamblia* can also be the cause of diarrhea. People who are lactose intolerant have trouble digesting lactose, a sugar included in milk and other dairy products, and are susceptible to diarrhea. There may be a lot of complications that a person may face due to diarrhea. The important side effect of this disease, which appears, particularly in newborn babies, young children and the elderly, is in the form of dehydration which happens when the human body loses electrolytes and too much liquid. Severe dehydration could be fatal and needs medical treatment. In this article, diarrhea disease dynamics are investigated mathematically.

From the earliest times of civilization, diarrheal illnesses have been reported, as demonstrated by the hieroglyphs found in the Ebers papyrus (3300 BC) and the Hearst papyrus (3300 BC), representing diarrhea and watery diarrhea separately [2]. All over the world, mortality and morbidity among children of every age group have been affected by diarrheal disease and malnutrition mostly in children under 5 years old also has been the cause of this disease. According to the report of the World Health Organization (WHO), Approximately 443,832 children die from diarrheal disease each year. This ratio makes diarrhea disease, the third leading cause of under 5 years of age children’s death every year [3]. Medical experts suggest safe drinking water, the use of effective sanitation and hand washing with clean soap, as the prevention measures. Diarrheal patients should the oral rehydration solution, usually known as ORS, which is a mixture of sugar, salt and clean water. Furthermore, a 10-to 14-day additional course of treatment consisting of dispersive zinc pills reduces the duration of diarrhea and improves its results. The history of applications of differential equations to model the biological, chemical reaction, ecological and competition systems goes back to Verhulst, Malthus, Lotka, and Volterra [4]. It is well-recognized that using differential equations to model natural phenomena, can be beneficial to analyze the systems understudy, for instance, it is commonly accepted that partial differential equations are extremely valuable to understanding population dynamics, the spread of infectious diseases, interactions between two or more species, and other biological phenomena [5–8]. Recently, numerous researchers investigated the systems of differential equations and described the behaviors of their solutions [9–16]. In [17], Akinola *et al*. examined immunity and the norovirus infection dynamic transmission model. It was discovered that even slight changes in the basic reproduction number can significantly impact the asymptomatic spread of norovirus. In the research work of Ardkaew and Tongkumchum, the mathematical model of diarrhea disease is studied with the application in control and prevention [18]. The model demonstrated the pattern formations of the dynamics of infantile diarrhea diseases together with the rotavirus or enterotoxigenic Escherichia coli. Olutimo and Williams examined a compartmental model (SITR), in which authors analyzed the dynamic effects of treatment on the transmission dynamics of diarrhea disease in the locality [19]. Cherry [20] assessed the management of the bovine viral diarrhea virus by using a mathematical model that revealed the infection dynamics. Other comparable studies on endemic diseases are constructed, determining the number of confirmed cases, deaths, and recoveries to manage the disease in the presence of treatment and vaccination using a similar model [21–23]. Naveed *et al*. [24] studied an epidemiological delayed model of diarrhea disease with treatment effects. The artificial delay parameter used in this model is defined with a saturated incidence rate. In this article, the authors verified the positivity and boundedness of the continuous model. The sensitivity of the parameters, as well as, the local and global stability of the equilibrium points are also proved by the Routh Hurwitz criterion and Lyapunov function, respectively.

In this study, a mathematical model of diarrhea disease with spatial diffusion is investigated analytically and numerically.

In Section 2, the model with suitable assumptions is described. The results regarding positivity and boundedness are also developed analytically in Section 3. Global stability of the disease-free equilibrium and endemic equilibrium points are also verified by using the Lyapunov function technique in Section 4. Section 5 comprises numerical analysis for the model under study. In this context, a nonstandard finite difference scheme is applied to the proposed model. Some structural properties of the numerical technique are proved in this section, for instance, positivity, consistency, boundedness and nonlinear stability with the help of Gronwall’s inequality. Section 6 consists of the simulations of the state variables and a productive discussion about the graphical behaviors of the variables involved in the model. In Section 7 some concluding remarks are described to understand the achievements of the goal in this section.

## 2 Model with its important threshold

Naveed M. *et al* [24] studied numerically, an epidemic model of diarrhea disease with a delay factor which is given as follows:

$$S^{\prime }(t)=\Lambda +\alpha_{1}R(t)-\alpha_{2}S(t)-(\frac{\beta_{1}I(t)}{1+\alpha I(t)}+\frac{\beta_{2}\alpha_{3}T(t)}{1+\alpha T(t)})S(t),$$ (1)

$$I^{\prime }(t)=(\frac{\beta_{1}I(t)}{1+\alpha I(t)}+\frac{\beta_{2}\alpha_{3}T(t)}{1+\alpha T(t)})S(t)-(\alpha_{2}+\alpha_{4})I(t),$$ (2)

$$T^{\prime }(t)=P\alpha_{4}I(t)-(\alpha_{2}+\alpha_{5})T(t),$$ (3)

$$R^{\prime }(t)=(1-P)\alpha_{4}I(t)+\alpha_{5}T(t)-(\alpha_{2}+\alpha_{1})R(t),$$ (4)

for t≥0.

In the above system, four sub-populations are involved named susceptible *S*(*t*), infected *I*(*t*), treated *T*(*t*), and recovered *R*(*t*). Assuming that all these state variables remain nonnegative at every value of time *t*. Other nonnegative parameters used in the model are described in Table 1.

**Table 1:** Parameters description.

Notations	Description	values/Day	Sources
Λ	Recruitment rate	0.5	[18]
$\alpha_{1}$	Rate of transmission again from recovered to infected	0.8	[18]
$\alpha_{2}$	Natural death rate which is same in each compartment	0.5	[18]
$\beta_{1}$	Contact rate	1.00093(DFE) 2.00093(DEE)	[18]
$\beta_{2}$	Saturation treatment rate	1.0031(DFE) 2.0031(DEE)	[18]
α_3_	Enhancement factor	0.2	[18]
α	Educated adjustment	0.012	[18]
$\alpha_{4}$	Rate of transmission from infected to treated	0.7	[18]
*P*	The probability of the infected persons that may be a part of *R*(*t*) or *T*(*t*)	0.04	[18]
$\alpha_{5}$	Transmission rate from treated to recovered	0.9	[18]
*N*	Total population		

In the current study, the above model is analyzed more generically. To capture the spatial dynamics, the proposed model is formed by incorporating the diffusion term that changes the whole scenario of the study. The diffusion term in each compartment represents the random movement of persons in different neighboring locations that reflects the spatial spread of the disease along a single spatial dimension. Mathematical analysis of spatio-temporal models helps us to understand the dynamics of a disease at any time *t* over the space *x*, optimal control, and investigation strategies. The diffusive model is designed as follows:

$$S_{t}(x,t)=\Lambda +\alpha_{1}R(x,t)-\alpha_{2}S(x,t)-\;\;\;\;(\frac{\beta_{1}I(x,t)}{1+\alpha I(x,t)}+\frac{\beta_{2}\alpha_{3}T(x,t)}{1+\alpha T(x,t)})S(x,t)+d_{1}S_{xx}(x,t),$$ (5)

$$I_{t}(x,t)=(\frac{\beta_{1}I(x,t)}{1+\alpha I(x,t)}+\frac{\beta_{2}\alpha_{3}T(x,t)}{1+\alpha T(x,t)})S(x,t)-(\alpha_{2}+\alpha_{4})I(x,t)+\;d_{2}I_{xx}(x,t),$$ (6)

$$T_{t}(x,t)=P\alpha_{4}I(x,t)-(\alpha_{2}+\alpha_{5})T(x,t)+d_{3}T_{xx}(x,t),$$ (7)

$$R_{t}(x,t)=(1-P)\alpha_{4}I+\alpha_{5}T(x,t)-(\alpha_{1}+\alpha_{2})R(x,t)+d_{4}R_{xx}(x,t),$$ (8)

for t≥0,τ≤t.

In the model (5)-(8), the diffusion reflects the spatial movements of individuals across the spatial domain. The diffusion coefficients $d_{1},d_{2},d_{3}$ and *d*_4_ are associated with the rate of mobilization of susceptible, infected, treated and recovered respectively. The coefficients describe the natural random spread and movements of the people that may be due to different factors, for instance, traveling, migration, interactions of the people in the defined environment, etc. To involve the diffusion coefficients in the corresponding compartmental equation of the model understudy, it is important to make some suitable assumptions, which may be as follows:

(i) The random movement of the people obeys the standard diffusion procedure in which people move from high-concentration to low-concentration areas, i.e., the movement of the individuals is from high-density areas to low-density areas.
(ii) All the diffusion coefficients $d_{1},d_{2},d_{3}$ and *d*_4_ are considered as constants that reveal uniformity in the spatial mobilization of the people.
(iii) The diffusion rate corresponding to each compartment is considered different, for instance, the movement of the infected individuals may be slow as compared to the susceptible due to the symptoms or some isolation policies implemented by the authorities.

The mathematical study of epidemic models enables us to predict the dynamic behavior of the disease which can help us to suggest control strategies for the disease. The model understudy can be modified by incorporating various factors, such as airborne, vector-born, or other transmission diseases with direct contact by rearranging the transmission terms. By involving the incubation period (exposed) compartment in the current model, the study becomes more effective with a significant incubation stage, e.g., COVID-19. The transmission rates between the compartmental population can be modified in different ways and the performance of specific pathogen treatment with recovery rates can be included.

### 2.1 Model assumptions

It is important to make some assumptions that will help to prove some important results regarding the model. Suppose that Ω is a bounded and nonnegative subset of the set *R*^ + ^ with smooth boundary ∂Ω. Also, $\overset{―}{\Omega }$ represents its closure and for any *T* > 0, [0, *T*] is the temporal domain. Then we can assume that Σ=Ω × [0,T] be a nonempty set of continuous functions. The state variables S,I,T,R∈Σ, sufficiently smooth functions in $C^{2}(\Sigma )$, are considered to be the *S*(*x*, *t*), *I*(*x*, *t*), *T*(*x*, *t*), and *R*(*x*, *t*) respectively. Due to the population dynamic model, the state variables should always be nonnegative, i.e., S,I,T,R≥0 in Σ. Also, let the diffusion coefficients $d_{1},d_{2},d_{3},d_{4}$ included in the system, be nonnegative.

The nonnegative initial and boundary conditions for the system (5)-(8) are supposed to be


$$S(x,0)=S_{0}(x)\geq 0,I(x,0)=I_{0}(x)\geq 0,T(x,0)=T_{0}(x)\geq 0,$$

$$R(x,0)=R_{0}(x)\geq 0,\text{ for }x\in \overset{―}{\Omega },$$ (9)

with

$$\frac{\partial S}{\partial \varrho }=\frac{\partial I}{\partial \varrho }=\frac{\partial T}{\partial \varrho }=\frac{\partial R}{\partial \varrho }=0,\text{ for all }x\in \partial \Omega ,\text{ for }t>0,$$ (10)

where ϱ stands for the outward normal direction and $\frac{\partial S}{\partial \varrho },\frac{\partial I}{\partial \varrho },\frac{\partial T}{\partial \varrho }$ and $\frac{\partial R}{\partial \varrho }$ define the outward flux.

## 3 About the prescribed model

Before starting the analysis of the SITR model, it is better to recall some important features and assumptions of the model equations. This discussion can be beneficial for the readers to understand the dynamics of the disease. The model presented in this article is a SITR model as defined in the previous section, which incorporates several important traits such as spatial diffusion, saturation treatment rates, enhancement factors, educated adjustment, and the dynamic interaction between treatment and recovered compartments. These features improve the ability to understand the transmission dynamics of this disease. In the presence of these salient features, the proposed model becomes more valuable for both short-term and long-term predictions. The spatial diffusion included in the model equations is the spread of disease in different locations. By designing a mathematical model that reflects the spread of disease between different locations, the model enables the prediction of areas with new cases of disease and the identification of necessary measures in these areas. The model having a saturated treatment rate reflects the ability to know how the healthcare system overcomes the disease by treating a limited number of individuals. The incorporation of two important quantities, educated adjustments and enhancement factors enable the mathematical model to describe the effects of the campaigns launched by the local Public Health Departments, such as vaccination, keeping hands clean, etc. The compartmental models of disease dynamics depict the compartmental transition of people, i.e., how individuals move from the susceptible compartment to the infected class and infection class to the treated class and treated to the recovered compartment. The compartmental model shows how people move from being infected to treated and from treated to recovered, which is essential for estimating the rate at which an outbreak may slow down. The steady-state analysis of the SITR epidemic model predicts the dynamic of the disease, i.e., whether the disease will die out or persist endemic. The transition of recovered people to the susceptible class again reflects the beginning of the outbreak of the disease. The incorporation of the reinfection factor in the infectious disease model makes the model more realistic and can help the Public Health Departments in long-term planning. The education impact over time included in the model helps to have a long-term effect on the dynamics of the disease.

On the other hand, there are some difficulties that one can face in analyzing the epidemic model like the SITR model. The collection of precise real data about parameters, for instance, saturated treatment rate, awareness factor and enhancement factor is important for short-term prediction but it is very hard to collect and then estimate them at the beginning of the outbreak. The present number of susceptible, infected, treated and recovered people have a key role in predicting a disease (based on the current model). Better prediction depends on the true initial values of these quantities. Errors can occur due to uncertainty in these values. Most of the parameters, such as treatment rate, enhancement factor, and awareness remain constant for the study of the SITR model in Long-term predictions. However, these values can vary according to the situation due to the changes in health policies, medical treatment, etc.

The present study is limited to demonstrating the dynamics of the disease by incorporating the diffusion in one dimension. This choice of one-dimensional diffusion in each compartment of the prescribed model allows for a brief and clear interpretation of the findings and reduces the computational complexities. This assumption just allows us for the numerical and analytical study. While the real scenario of the dynamics of the disease can be visualized in the two or three-dimensional space domains. To overcome this challenge, future work could extend and generalize the model understudy to include diffusion in two or three dimensions that can provide us with more realistic situations and demonstration of the spatial dynamics of disease.

After the detailed discussion of the model, the quantitative analysis of the model is presented in the next subsection.

### 3.1 Steady states for the system

In the study of dynamical systems, steady states (equilibrium points) play a vital role. The steady state of a dynamical system is the state at which there is no change over time. Likewise, in a population model of infectious disease, the rate of change of infected persons becomes zero over a particular time at the equilibrium points. According to the theory of infectious diseases, there are always some states at which the disease transmission has stopped and infected individuals are either recovered or there is no increase or decrease in number. The stability of the equilibrium indicates the future behavior of the disease and whether and when the disease will die out. To find the equilibrium point mathematically, each equation of the system equates to zero and gets a system of algebraic equations. After solving these equations simultaneously, we get the values of the state variables. These values are called the equilibrium points for the system under study.

To discuss the equilibria of the system, all the rates of change involved in the equations keep zero, that is,

$$\Lambda +\alpha_{1}R-\alpha_{2}S-(\frac{\beta_{1}I}{1+\alpha I}+\frac{\beta_{2}\alpha_{3}T}{1+\alpha T})S=0,\;(\frac{\beta_{1}I}{1+\alpha I}+\frac{\beta_{2}\alpha_{3}T}{1+\alpha T})S-(\alpha_{2}+\alpha_{4})I=0,\;P\alpha_{4}I-(\alpha_{2}+\alpha_{5})T=0,\;(1-P)\alpha_{4}I+\alpha_{5}T-(\alpha_{2}+\alpha_{1})R=0,$$ (11)

Disease free equilibrium *E*_*df*_ of the model is defined as:

$$E_{df}\equiv (S_{0},I_{0},T_{0},R_{0})=(\frac{\Lambda }{\mu },0,0,0,0).$$ (12)

Also, the endemic equilibrium point

$$E_{e}\equiv (S^{*},I^{*},T^{*},R^{*}),$$ (13)

can be calculated as


$$A=\frac{P\alpha_{4}}{\alpha_{2}+\alpha_{5}},\;B=\alpha_{4}\alpha_{2}-\alpha_{2}P\alpha_{4}+\alpha_{4}\left(\alpha_{2}+\alpha_{1})(\alpha_{2}+\alpha_{5}\right),\;C=\frac{\Lambda }{\alpha_{2}},$$

with


$$D=[\frac{\alpha_{1}(\alpha_{2}\alpha_{4}\;-\;\alpha_{2}P\alpha_{4}\;+\;\alpha_{5}\alpha_{4})\;-\;(\alpha_{2}\;+\;\alpha_{4})(\alpha_{2}\;+\;\alpha_{4})(\alpha_{2}\;+\;\alpha_{5})}{\alpha_{2}(\alpha_{2}\;+\;\alpha_{1})(\alpha_{2}\;+\;\alpha_{5})}],S^{*}=C\;+\;DI^{*},$$

$$T^{*}=AI^{*},\;R^{*}=CB,$$

$$I^{*}=\frac{-(\Delta_{5}-\Delta_{2})+\sqrt{\left(\Delta_{5}-\Delta_{2}{)}^{2}-4(\Delta_{4}-\Delta_{1})(\Delta_{6}-\Delta_{3}\right)}}{2(\Delta_{4}-\Delta_{1})},$$

in which


$$\Delta_{1}=(\alpha A\beta_{1}+\alpha A\alpha_{3}\beta_{2})D,\;\;\;\;\;\;\;\;\;\;\;\;\;\Delta_{2}=(\beta_{1}+\beta_{2}\alpha_{3}A)D,$$

$$\Delta_{3}=(\beta_{1}+\beta_{2}\alpha_{3}A+kA\beta_{1}+\alpha A\alpha_{3}\beta_{2})C,\Delta_{4}=\alpha A(\alpha_{2}+\alpha_{4}),$$

$$\Delta_{5}=(\alpha_{2}+\alpha_{4})(\alpha +\alpha A),\;\;\;\;\;\;\;\;\;\;\;\;\;\;\;\;\;\;\;\;\;\;\Delta_{6}=(\alpha_{2}+\alpha_{4}).$$

The Basic reproductive number *R*_0_ is defined as [24]

$$R_{0}=\frac{\Lambda (\beta_{1}\alpha_{2}+\beta_{2}\alpha_{5}+P\alpha_{4}\beta_{2}\alpha_{3})}{\alpha_{2}(\alpha_{2}+\alpha_{5})(\alpha_{2}+\alpha_{4})}.$$ (14)

### 3.2 Analysis of the model

To obtain the result regarding the positivity of the state variables of the model (5)-(8), the following lemma helps to verify.

**Lemma 3.1.** *[25] Suppose that Q be a sufficiently smooth function in the function space ℱ defined by*


$$ℱ=C^{2,1}(\Sigma \times (0,T])\cap C(\overset{―}{\Sigma }\times [0,T]),$$

*that obeys the differential inequality*


$$Q_{t}-b\Delta Q\geq y(x,t)Q,\;x\in \Sigma ,0<t\leq T,Q\in ℱ,$$

*together with the boundary and initial conditions*


$$Q_{\eta }\geq 0,\;\forall \;x\in \partial \Sigma ,\;0<t\leq T,$$

*and also,*


$$Q(x,0)\geq 0,\;\forall x\in \overset{―}{\Sigma },$$

*in which*


$$y(x,t)\in C(\overset{―}{\Sigma }\times [0,T]).$$

*Then Q(x,t)≥0 on $\overset{―}{\Sigma }\times [0,T]$. Also, Q(x,t) > 0 or *Q* = 0 in Σ×[0,T].*

Now, by using the aforementioned lemma 3.1, the following theorem is possible to establish.

**Theorem 3.1.** (Non-negativity) Suppose that the initial conditions for the system (5)-(8) are considered to be positive and all the assumptions of subsection 2.1, hold, then the system possesses the positive solution.

Proof: Assuming the vector (S, I, T, R) are regarded as the solutions to (5)-(8), where


$$S,I,T,R\in ℱ=C(\overset{―}{\Sigma }\times [0,T_{max}))\cap C^{2,1}(\Sigma \times [0,T_{max})).$$

Then for any $t^{*}\in (0,T_{max})$, by taking the equation of (5), we get


$$S_{t}-d_{1}S_{xx}=-\{\alpha_{2}+(\frac{\beta_{1}I}{1+\alpha I}+\frac{\beta_{2}\alpha_{3}T}{1+\alpha T})\},\;0<t<t^{*}.$$

Since the total population is bounded at every point in the space $\Sigma \times [0,t^{*}]$, it follows that


$$-\{\alpha_{2}+(\frac{\beta_{1}I}{1+\alpha I}+\frac{\beta_{2}\alpha_{3}T}{1+\alpha T})\}$$

must also be bounded. Consequently, all the conditions outlined in the preceding Lemma 3.1 are met.

So,


$$S>0\text{ in }\Sigma \times (0,t^{*}].$$

On combining the equation (6) with Lemma 3.1, we can exhibit the positivity of the variable *I* because,


$$I_{t}-d_{2}I_{xx}(x,t)=-(\alpha_{2}+\alpha_{4})I,\;,0<t<t^{*}.$$

Due to the boundedness of the population at every point of $\Lambda \times [0,t_{*}]$, the factor $-(\alpha_{2}+\alpha_{4})$ is also bounded, so all requirements for Lemma 3.1 are accomplished. So,


$$I>0\text{ in }\Sigma \times (0,t^{*}].$$

Also, the positivity of *T* can be proved from the equation (7) by using Lemma 3.1 because,


$$T_{t}-d_{3}T_{xx}=-(\alpha_{2}+\alpha_{5})T,\;,0<t<t^{*},$$

we have


$$T>0\text{ in }\Sigma \times (0,t^{*}].$$

In the end, from the last equation (8) of the model


$$R_{t}=(1-P)+\alpha_{5}T-(\alpha_{2}+\alpha_{1})R+d_{3}R_{xx},\;,0<t<t^{*},$$

we get


$$R>0\text{ in }\Sigma \times (0,t^{*}].$$

Now, since $t^{*}\in (0,T_{max})$ is an arbitrary so, it can be concluded that all state variables *S*, *I*, *T* and *R* are positive in the whole domain $\Sigma \times [0,T_{max})$. ◻

**Theorem 3.2.** *(Boundedness) Suppose that the vector (S, I, T, R) be the solution of the system (*5*)-(*8*), where $S,I,T,R\in C(\overset{―}{\Sigma }\times [0,T_{max}))\cap C^{2,1}(\Sigma \times [0,T_{max}))$. Then the system (*5*)-(*8*) has uniformly bounded solution on $\overset{―}{\Sigma }$, given that the condition (*9*)-(*10*) satisfies.*

*Proof:* By adding equations in (5)-(8), we get


$$\frac{\partial S}{\partial t}+\frac{\partial I}{\partial t}+\frac{\partial T}{\partial t}+\frac{\partial R}{\partial t}-d_{1}\frac{\partial^{2}S}{\partial x^{2}}-d_{2}\frac{\partial^{2}I}{\partial x^{2}}-d_{3}\frac{\partial^{2}T}{\partial x^{2}}-d_{4}\frac{\partial^{2}R}{\partial x^{2}}$$

$$=\Lambda -\alpha_{2}(S+I+T+R).$$

Integrating both sides, we have

$$\int_{\Sigma }(\frac{\partial S}{\partial t}+\frac{\partial I}{\partial t}+\frac{\partial T}{\partial t}+\frac{\partial R}{\partial t}-d_{1}\frac{\partial^{2}S}{\partial x^{2}}-d_{2}\frac{\partial^{2}I}{\partial x^{2}}-d_{3}\frac{\partial^{2}T}{\partial x^{2}}-\;d_{4}\frac{\partial^{2}R}{\partial x^{2}})dx=\int_{\Sigma }(\Lambda -\alpha_{2}(S+I+T+R))dx.$$ (15)

According to the Green’s formula, we recall


$$d_{1}\int_{\Sigma }\frac{\partial^{2}S}{\partial x^{2}}dx=d_{1}\int_{\partial \Sigma }\frac{\partial S}{\partial \varrho }dx,\;d_{2}\int_{\Sigma }\frac{\partial^{2}I}{\partial x^{2}}dx=d_{2}\int_{\partial \Sigma }\frac{\partial I}{\partial \varrho }dx,$$

$$d_{3}\int_{\Sigma }\frac{\partial^{2}T}{\partial x^{2}}dx=d_{3}\int_{\partial \Sigma }\frac{\partial T}{\partial \varrho }dx,\;d_{4}\int_{\Sigma }\frac{\partial^{2}R}{\partial x^{2}}dx=d_{4}\int_{\partial \Sigma }\frac{\partial R}{\partial \varrho }dx.$$

By using the Neumann conditions from (10), it is known


$$\frac{\partial M}{\partial \varrho }=\frac{\partial S}{\partial \varrho }=\frac{\partial L}{\partial \varrho }=\frac{\partial I}{\partial \varrho }=\frac{\partial R}{\partial \varrho }=0,\;\text{for all}\;x\in \partial \Sigma ,\;t>0.$$

Equation (15) gives


$$\int_{\Sigma }(\frac{\partial S}{\partial t}+\frac{\partial I}{\partial t}+\frac{\partial T}{\partial t}+\frac{\partial R}{\partial t})dx=\int_{\Sigma }(\Lambda -\alpha_{2}(S+I+T+R))dx,$$

$$\leq \int_{\Sigma }(\Lambda -\alpha_{2}(S+I+T+R))dx,$$

$$=\Lambda |\Sigma |-\beta \int_{\Sigma }(S+I+T+R)dx.$$ (16)

Letting $\int_{\Sigma }(S+I+T+R)dx=𝒜(t)$, equation (16) gives


$$\frac{d(𝒜(t))}{dt}\leq \Lambda |\Sigma |-\alpha_{2}𝒜(t).$$

It gives


$$0\leq 𝒜(t)\leq \frac{\Lambda }{\alpha_{2}}|\Sigma |+𝒜(0)e^{-\alpha_{2}t}.$$

So, $𝒜(t)\leq \text{max}\{𝒜(0),\frac{P\Sigma }{\Sigma }\}$,

in which


$$𝒜(0)=\int_{\Sigma }\{S(x,0)+I(x,0)+T(x,0)+R(x,0)\}dx,$$

$$\leq \int_{\Sigma }\|S(x,0)+I(x,0)+T(x,0)+R(x,0){\|}_{\infty }dx,$$

$$=\|S(x,0)+I(x,0)+T(x,0)+R(x,0){\|}_{\infty }|\Sigma |.$$

This gives us the boundedness of 𝒜(t) by Theorem 3.1 and equation (16), So,


$$||S+I+T+R{||}_{L^{1}(\Sigma )}=\int_{\Sigma }|S+I+T+R|dx,$$

$$=\int_{\Sigma }(S+I+T+R)dx,$$

$$\leq \text{max}\{||S(x,0)+I(x,0)+T(x,0)+R(x,0){||}_{\infty }\Sigma ,\frac{\Lambda |\Sigma |}{\alpha_{2}}\}.$$

Let $\zeta =\text{max}\{||S(x,0)+I(x,0)+T(x,0)+R(x,0){||}_{\infty }\Sigma ,\frac{\Lambda |\Sigma |}{\alpha_{2}}\}$, then


$$\int_{\Sigma }(S+I+T+R)dx\leq \zeta .$$

According to the Theorem 3.1 of [26], there exists a positive number $\zeta^{*}$, such that


$$||S+I+T+R{||}_{L^{\infty }(\Sigma )}\leq \zeta^{*}.$$

So, it can be concluded that *S*(*x*, 0), *I*(*x*, *t*), *T*(*x*, *t*), *R*(*x*, *t*) are uniformly bounded on $\overset{―}{\Sigma }$, hence, bounded. ◻

## 4 Stability analysis

The stability of a dynamical system plays a significant role in understanding the behavior of its solution. It describes how the system’s solutions behave near its equilibrium point, i.e., due to a small perturbation, whether the trajectories of the solution remain close to the equilibrium point of the system or show divergence. In the study of epidemiology, the stability of the infectious disease model, especially, a diffusive model, describes the convergence of its solution towards the disease-free and equilibrium points over a finite time domain under certain conditions on the basic reproductive number, *R*_0_. Stability analysis of an epidemic model of infectious disease depicts the spreading behavior of the disease. It helps us identify if the epidemic’s spread demonstrates chaotic or unbounded behavior or stabilizes (e.g., approaches an equilibrium point).

In the study of dynamical systems, stability and boundedness perform a critical role in guaranteeing the reliability of the approximate solutions, particularly, for disease dynamic models over finite time intervals (say) [0,*T*]. The current research also presents the stability of a numerical scheme over a finite time interval. To demonstrate this, the stability of the proposed NSFD method over a finite time domain t∈[0,T] is analyzed in this section, where *T* is the maximum time required. The NSFD scheme is constructed such that the positivity and boundedness of the state variables *S*, *I*, *T* and *R* are preserved within the time interval [0, *t*]. In this respect, it is assumed that S(x,t)+I(x,t)+T(x,t)+R(x,t)≤N(x,0), for all t∈[0,T].

In the next section global stability of the diffusive model is presented by using the Lyapunov function for which it is proved that the Lyapunov function is non-increasing.

### 4.1 Stability of the model

**Theorem 4.1.** *[24] The disease-free equilibrium* $E_{df}=(S_{0},I_{0},T_{0},R_{0})$ *for the model (*1*)-(*4*) (without diffusion) is globally asymptotically stable (GAS), if* *R*_*0*_*<1.*

**Theorem 4.2.** *If the basic reproduction number* *R*_*0*_*<1, the disease-free equilibrium* $\left(E_{df}(S_{0},I_{0},T_{0},R_{0})\right)$ *for the system (*5*)-(*8*) is globally asymptotically stable (GAS).*

*Proof:* Let us chose a Lyapunov function for the model (1)-(4) defined in Theorem 4 of [24]


$$V_{1}=(S-S_{0}-S_{0}ln\frac{S}{S_{0}})+I+T+R.$$

Now, consider a Lyapunov function for the model (4)-(8) as following


$$V_{1}=\int_{\Sigma }V_{1}(g(x,t))dx,$$

where


g(x,t)=(S(x,t),I(x,t),T(x,t),R(x,t)).

Then


$$\frac{dV}{dt}=\int_{\Sigma }grad_{g}V_{1}.\frac{\partial g}{\partial t}dx,$$

$$=\int_{\Sigma }(1-\frac{S_{0}}{S},1,1,1).(S^{\prime }+d_{1}\Delta S,I^{\prime }+d_{2}\Delta I,T+\;d_{3}\Delta T,R^{\prime }+d_{4}\Delta R)dx,$$

$$=\int_{\Sigma }\{(1-\frac{S_{0}}{S})S^{\prime }+I^{\prime }+T^{\prime }+R^{\prime }\}dx+d_{1}\int_{\Sigma }(1-\frac{S_{0}}{S})\Delta Sdx+\;d_{2}\int_{\Sigma }\Delta Idx+d_{3}\int_{\Sigma }\Delta Tdx+d_{4}\int_{\Sigma }\Delta Rdx,$$

$$=\int_{\Sigma }\frac{dV_{1}}{dt}dx+d_{1}\int_{\Sigma }\Delta Sdx-d_{1}S_{0}\int_{\Sigma }\frac{\Delta S}{S}dx+d_{2}\int_{\Sigma }\Delta Idx+d_{3}\int_{\Sigma }\Delta Tdx+\;d_{4}\int_{\Sigma }\Delta Rdx,$$

$$=\int_{\Sigma }\frac{dV_{1}}{dt}dx-d_{1}S_{0}\int_{\Sigma }\frac{|\Delta S{|}^{2}}{S^{2}}dx-d_{4}S_{0}\int_{\Xi }\frac{|\Delta S{|}^{2}}{S^{2}}dx,$$

where Green’s formulas give


$$d_{1}\int_{\Sigma }\Delta Sdx=d_{1}\int_{\partial }\Sigma \frac{\partial S}{\partial \varrho }dx,$$

$$d_{2}\int_{\Sigma }\Delta Idx=d_{2}\int_{\partial }\Sigma \frac{\partial I}{\partial \varrho }dx,$$

$$d_{3}\int_{\Sigma }\Delta Tdx=d_{3}\int_{\partial }\Sigma \frac{\partial T}{\partial \varrho }dx,$$

$$d_{4}\int_{\Sigma }\Delta Rdx=d_{4}\int_{\partial }\Sigma \frac{\partial R}{\partial \varrho }dx,$$

and


$$\int_{\Sigma }\frac{\Delta S}{S}dx=\int_{\Sigma }\frac{|\Delta S{|}^{2}}{S^{2}}dx.$$

Also, by the conditions (9)-(10)


$$\frac{\partial S}{\partial \varrho }=\frac{\partial I}{\partial \varrho }=\frac{\partial T}{\partial \varrho }=\frac{\partial R}{\partial \varrho }=0,\;\text{ for all }x\in \partial \Sigma ,t>0.$$

Now, the verification of global stability of the system (1)-(4) implies


$$\frac{dV_{1}}{dt}<0.$$

Together with all above results, we obtain


$$\frac{dV}{dt}<0,$$

that guarantees the global stability for the diffusive model (5)-(8). ◻

**Theorem 4.3.** *[24] The endemic equilibrium* $E_{e}=(S^{*},I^{*},T^{*},R^{*})$ *for the model (*1*)-(*4*) (without diffusion) is globally asymptotically stable (GAS), if R*_*0*_*>1.*

**Theorem 4.4.** *For the reproduction number* *R*_*0*_*>1, the endemic equilibrium $E_{e}=(S^{*},I^{*},T^{*},R^{*})$ for the model (*5*)-(*8*) (with diffusion) is globally asymptotically stable (GAS).*

*Proof:* As a Lyapunov function for the model (1)-(4) in [24],


$${𝒯}_{1}=(S-S^{*}-S^{*}ln\frac{S}{S^{*}})+(I-I^{*}-I^{*}ln\frac{I}{I^{*}})+(T-T^{*}-T^{*}ln\frac{T}{T^{*}})+\;(R-R^{*}-R^{*}ln\frac{R}{R^{*}}).$$

Now, consider a Lyapunov function as given below


$$𝒯=\int_{\Sigma }{𝒯}_{1}(g(x,t))dx,$$

where


g(x,t)=(S(x,t),I(x,t),T(x,t),R(x,t)).

Then


$$\;\;\frac{d𝒯}{dt}=\int_{\Sigma }grad_{g}{𝒯}_{1}.\frac{\partial g}{\partial t}dx,$$

$$=\int_{\Sigma }(1-\frac{S^{*}}{S},1-\frac{I^{*}}{I},1-\frac{T^{*}}{T},1-\frac{R_{*}}{R}).(S^{\prime }+d_{1}\Delta S,I^{\prime }+\;d_{2}\Delta I,T^{\prime }+d_{3}\Delta T,R^{\prime }+d_{5}\Delta R)dx,$$

$$=\int_{\Sigma }\{(1-\frac{S^{*}}{S})S^{\prime }+1-\frac{I^{*}}{I})I^{\prime }+(1-\frac{T^{*}}{T})T^{\prime }+\;(1-\frac{R_{*}}{R})R^{\prime }\}dx+d_{1}\int_{\Sigma }(1-\frac{S^{*}}{S})\Delta Sdx+d_{2}\int_{\Sigma }(1-\frac{I^{*}}{I})\Delta Idx+\;d_{3}\int_{\Sigma }(1-\frac{T_{*}}{T})\Delta Tdx+d_{4}\int_{\Sigma }(1-\frac{R^{*}}{R})\Delta Rdx,$$

$$=\int_{\Sigma }\frac{d{𝒯}_{1}}{dt}dx+d_{1}\int_{\Sigma }\Delta Sdx-d_{1}S^{*}\int_{\Sigma }\frac{\Delta S}{S}dx+d_{2}\int_{\Sigma }\Delta Idx-d_{2}I^{*}\int_{\Sigma }\frac{\Delta I}{I}dx+\;d_{3}\int_{\Sigma }\Delta Idx-d_{3}I^{*}\int_{\Sigma }\frac{\Delta T}{T}dx+d_{4}\int_{\Sigma }\Delta Rdx-d_{3}R^{*}\int_{\Sigma }\frac{\Delta R}{R}dx,$$

$$=\int_{\Sigma }\frac{d{𝒯}_{1}}{dt}dx-d_{1}S^{*}\int_{\Sigma }\frac{|\Delta S{|}^{2}}{S^{2}}dx-d_{2}I^{*}\int_{\Sigma }\frac{|\Delta I{|}^{2}}{I^{2}}dx-d_{3}L_{*}\int_{\Sigma }\frac{|\Delta T{|}^{2}}{T^{2}}dx-\;d_{4}R_{*}\int_{\Sigma }\frac{|\Delta R{|}^{2}}{R^{2}}dx,$$

where Green’s formulas give


$$d_{1}\int_{\Sigma }\Delta Sdx=d_{1}\int_{\partial }\Sigma \frac{\partial S}{\partial \varrho }dx,$$

$$d_{2}\int_{\Sigma }\Delta Idx=d_{2}\int_{\partial }\Sigma \frac{\partial I}{\partial \varrho }dx,$$

$$d_{3}\int_{\Sigma }\Delta Tdx=d_{3}\int_{\partial }\Sigma \frac{\partial T}{\partial \varrho }dx,$$

$$d_{4}\int_{\Sigma }\Delta Rdx=d_{4}\int_{\partial }\Sigma \frac{\partial R}{\partial \varrho }dx,$$

and


$$\int_{\Sigma }\frac{\Delta S}{S}dx=\int_{\Sigma }\frac{|\Delta S{|}^{2}}{S^{2}}dx,\int_{\Sigma }\frac{\Delta I}{I}dx=\int_{\Sigma }\frac{|\Delta I{|}^{2}}{I^{2}}dx,\int_{\Sigma }\frac{\Delta T}{T}dx=\int_{\Sigma }\frac{|\Delta T{|}^{2}}{T^{2}}dx,\;\int_{\Sigma }\frac{\Delta R}{R}dx=\int_{\Sigma }\frac{|\Delta R{|}^{2}}{R^{2}}dx.$$

Also, by the condition (9)-(10)


$$\frac{\partial S}{\partial \varrho }=\frac{\partial I}{\partial \varrho }=\frac{\partial T}{\partial \varrho }=\frac{\partial R}{\partial \varrho }=0,\;\text{ for all }\;x\in \partial \Sigma ,t>0.$$

The guarantee of global stability for the simple model (1)-(4) refers that


$$\frac{d{𝒯}_{1}}{dt}<0.$$

Together with all above results, we obtain


$$\frac{d𝒯}{dt}<0,$$

which is the fulfillment of the criteria for requirements of the global stability for the diffusive model (5)-(8). ◻

## 5 Numerical computations and investigation

Let us suppose that [0, *a*], [0, *b*] be the spatial and temporal domain for the model (5)-(8) and *r*, *s* be a pair of natural numbers. Also, suppose that $\lambda =\frac{a}{r}$ and $\rho =\frac{b}{s}$ represent the partition norm for the partitions of the intervals [0, *a*] and [0, *b*] respectively. Moreover, we can define $x_{i}=i\lambda$ and $t_{n}=n\rho$ for i∈{1,2,···,r} and n∈{1,2,···,s}. suppose that $S_{i}^{n}$, $I_{i}^{n}$, $T_{i}^{n}$, and $R_{i}^{n}$ be the approximations corresponding to the exact values $S(x_{i},t_{n})$, $I(x_{i},t_{n})$, $T(x_{i},t_{n})$ and $R(x_{i},t_{n})$ respectively at the mesh point (iλ,nρ), where i∈{1,2,···,r} and n∈{1,2,···,s}. Next, if, *K* represents any of the values *S*, *I*, *T* and *R*, then, we have


$$K^{n}=(K_{0}^{n},K_{1}^{n},\cdot \cdot \cdot ,K_{r}^{n}),\;n\in 0,1,2,\cdot \cdot \cdot ,s.$$

The discretization of the system (5)-(8) can be made by applying the linear discrete operators given below [27]

$$\delta_{t}K_{i}^{n+1}=\frac{K_{i}^{n+1}-K_{i}^{n}}{\rho },$$ (17)

$$\delta_{x}K_{i}^{n+1}=\frac{K_{i}^{n+1}-K_{i-1}^{n+1}}{\lambda },$$ (18)

$$\delta_{xx}K_{i}^{n+1}=\frac{K_{i+1}^{n+1}-2K_{i}^{n+1}+K_{i-1}^{n+1}}{\lambda^{2}},$$ (19)

where i∈{0,1,2,···,r} and n∈{0,1,2,···,s}. The discrete operators defined in Eqs. (17)-(18) representing the discretizations of the partial derivatives with respect to *t* and *x* respectively. While Eq. (19) approximates the double derivative with respect to *x*. All these approximations give the values of the time and space derivatives at points $\left(x_{i},t_{n}\right)$ and $\left(x_{i},t_{n+1}\right)$.

After using these operators in the system (5)-(8), we get a discrete system of algebraic equations.

$$\delta_{t}S_{i}^{n+1}=\Lambda +\alpha_{1}R_{i}^{n}-\alpha_{2}S_{i}^{n+1}-(\frac{\beta_{1}I_{i}^{n}}{1+\alpha I_{i}^{n}}+\frac{\beta_{2}\alpha_{3}T_{i}^{n}}{1+\alpha T_{i}^{n}})S_{i}^{n+1}+\;d_{1}\delta_{xx}S_{i}^{n+1},$$ (20)

$$\delta_{t}I_{i}^{n+1}=(\frac{\beta_{1}I_{i}^{n}}{1+\alpha I_{i}^{n}}+\frac{\beta_{2}\alpha_{3}T_{i}^{n}}{1+\alpha T_{i}^{n}})S_{i}^{n}-(\alpha_{2}+\alpha_{4})I_{i}^{n+1}+d_{2}\delta_{xx}I_{i}^{n+1},$$ (21)

$$\delta_{t}T_{i}^{n+1}=P\alpha_{4}I_{i}^{n}-(\alpha_{2}+\alpha_{5})T_{i}^{n+1}+d_{3}\delta_{xx}T_{i}^{n+1},$$ (22)

$$\delta_{t}R_{i}^{n+1}=(1-P)\alpha_{4}I_{i}^{n}+\alpha_{5}T_{i}^{n}-(\alpha_{1}+\alpha_{2})R_{i}^{n+1}+d_{4}\delta_{xx}R_{i}^{n+1}.$$ (23)

Now, using the Mickens rules for descretization defined in [27] are used on the nonlinear term and time step variations. Simple calculations for (20)-(23) give

$$-\varepsilon_{1}S_{i-1}^{n+1}+\{1+\rho \alpha_{2}+\rho (\frac{\beta_{1}I_{i}^{n}}{1+\alpha I_{i}^{n}}+\frac{\beta_{2}\alpha_{3}T_{i}^{n}}{1+\alpha T_{i}^{n}})+2\varepsilon_{1}\}S_{i}^{n+1}-\;\varepsilon_{1}S_{i+1}^{n+1}=S_{i}^{n}+\rho \Lambda +\alpha_{1}\rho R_{i}^{n},$$ (24)

$$-\varepsilon_{2}I_{i-1}^{n+1}+(1+\rho (\alpha_{2}+\alpha_{4})+2\varepsilon_{2})I_{i}^{n+1}-\varepsilon_{2}I_{i+1}^{n+1}=I_{i}^{n}+\;\rho (\frac{\beta_{1}I_{i}^{n}}{1+\alpha I_{i}^{n}}+\frac{\beta_{2}\alpha_{3}T_{i}^{n}}{1+\alpha T_{i}^{n}})S_{i}^{n},$$ (25)

$$-\varepsilon_{3}T_{i-1}^{n+1}+\{1+\rho (\alpha_{2}+\alpha_{5})+2\varepsilon_{3}\}T_{i}^{n+1}-\varepsilon_{3}T_{i+1}^{n+1}=T_{i}^{n}+P\alpha_{4}I_{i}^{n},$$ (26)

$$-\varepsilon_{4}R_{i-1}^{n+1}+\{1+\rho (\alpha_{1}+\alpha_{2})+2\varepsilon_{4}\}R_{i}^{n+1}-\varepsilon_{4}R_{i+1}^{n+1}=R_{i}^{n}+\;\rho (1-P)\alpha_{4}I_{i}^{n}+\alpha_{5}\rho T_{i}^{n},$$ (27)

where $\varepsilon_{p}=\frac{\rho d_{p}}{\lambda^{2}}$, p=1,2,3,4 with i∈{1,2,···,r}, n∈{0,1,2,···,s}.

The initial and boundary condition have the discretized form

$$S_{i}^{0}=S_{0}(x_{i}),\;I_{i}^{\;0}=I_{0}(x_{i}),\;T_{i}^{\;0}=T_{0}(x_{i}),\;R_{i}^{0}=R_{0}(x_{i}),\;\text{for}\;i\in \{1,2,\cdot \cdot \cdot ,r\},\;\text{ and }\;\delta S_{1}^{n}=\delta I_{1}^{n}=\delta T_{1}^{\;n}=\delta R_{1}^{n}=0,\;\delta S_{r}^{n}=\delta I_{r}^{n}=\delta T_{r}^{n}=\delta R_{r}^{n}=0,\;\text{for}\;n\in \{0,1,2,\cdot \cdot \cdot ,s\}.$$ (28)

One of the most important features for a population models is the nonnegativity of the variables involved in it, as these variables represent the density of the population. In the numerical study of such models, the positivity property should also be maintained. The numerical technique that is used to find the approximate solution of the system, should also preserve the same properties possessed by the continuous model.

### 5.1 Positivity

For a population dynamical system, the positivity of the state variables plays a vital role. So it must be preserved after employing the numerical scheme on the model. The following theorem reflects the positivity property.

**Theorem 5.1.** *Assume that 𝒯,𝒰,𝒱 and 𝒲 be the positive real valued functions depending on x defined in the interval (0, L) then the system (*24*)-(*27*), with the supportive data (*28*), has a solution ∀ n > 0 and i > 0. Moreover, the solutions are positive.*

*Proof:* It is interesting to note that the left-hand sides of each equation in (24)-(27) are implicitly related, so, one can deduce it in the vector representation as:

$$𝒯S^{n+1}=S_{i}^{n}+\lambda^{2}\rho (\Lambda +\alpha R_{i}^{n}),$$ (29)

$$𝒰I^{n+1}=I_{i}^{n}+\rho (\frac{\beta_{1}I_{i}^{n}}{1+\alpha I_{i}^{n}}+\frac{\beta_{2}\alpha_{2}T_{i}^{n}}{1+\alpha T_{i}^{n}})S_{i}^{n},$$ (30)

$$𝒱T^{n+1}=T_{i}^{n}+P\alpha_{4}I_{i}^{n},$$ (31)

$$𝒲R^{n+1}=R_{i}^{n}+\rho (1-P)\alpha_{4}I_{i}^{n}+\alpha_{5}\rho T_{i}^{n},$$ (32)

in which 𝒯,𝒰,𝒱 and 𝒲 are defined as (r+1)×(r+1) matrices. By using the initial and boundary conditions (28), we can find the matrices 𝒯,𝒰,𝒱 and 𝒲. Then


$$𝒯=(\begin{array}{cccccccc} (\chi_{1}{)}_{0}^{n} & \chi_{2} & 0 & \cdots & \cdots & \cdots & \cdots & 0\\ \chi_{3} & (\chi_{1}{)}_{1}^{n} & \chi_{4} & \ddots & & & & \vdots \\ 0 & \chi_{3} & (\chi_{1}{)}_{2}^{n} & \chi_{4} & \ddots & & & \vdots \\ \vdots & \ddots & \ddots & \ddots & \ddots & \ddots & & \vdots \\ \vdots & & \ddots & \ddots & \ddots & \ddots & \ddots & \vdots \\ \vdots & & & \ddots & \chi_{3} & (\chi_{1}{)}_{r-2}^{n} & \chi_{4} & 0\\ \vdots & & & & \ddots & \chi_{3} & (\chi_{1}{)}_{r-1}^{n} & \chi_{4}\\ 0 & \cdots & \cdots & \cdots & \cdots & 0 & \chi_{3} & (\chi_{1}{)}_{r}^{n} \end{array}),$$

$$𝒰=(\begin{array}{cccccccc} (\nu_{1}{)}_{0}^{n} & \nu_{2} & 0 & \cdots & \cdots & \cdots & \cdots & 0\\ \nu_{3} & (\nu_{1}{)}_{1}^{n} & \nu_{4} & \ddots & & & & \vdots \\ 0 & \nu_{3} & (\nu_{1}{)}_{2}^{n} & \nu_{4} & \ddots & & & \vdots \\ \vdots & \ddots & \ddots & \ddots & \ddots & \ddots & & \vdots \\ \vdots & & \ddots & \ddots & \ddots & \ddots & \ddots & \vdots \\ \vdots & & & \ddots & \nu_{3} & (\nu_{1}{)}_{r-2}^{n} & \nu_{4} & 0\\ \vdots & & & & \ddots & \nu_{3} & (\nu_{1}{)}_{r-1}^{n} & \nu_{4}\\ 0 & \cdots & \cdots & \cdots & \cdots & 0 & \nu_{3} & (\nu_{1}{)}_{r}^{n} \end{array}),$$

$$𝒱=(\begin{array}{cccccccc} (\eta_{1}{)}_{0}^{n} & \eta_{2} & 0 & \cdots & \cdots & \cdots & \cdots & 0\\ \eta_{3} & (\eta_{1}{)}_{1}^{n} & \eta_{4} & \ddots & & & & \vdots \\ 0 & \eta_{3} & (\eta_{1}{)}_{2}^{n} & \eta_{4} & \ddots & & & \vdots \\ \vdots & \ddots & \ddots & \ddots & \ddots & \ddots & & \vdots \\ \vdots & & \ddots & \ddots & \ddots & \ddots & \ddots & \vdots \\ \vdots & & & \ddots & \eta_{3} & (\eta_{1}{)}_{r-2}^{n} & \eta_{4} & 0\\ \vdots & & & & \ddots & \eta_{3} & (\eta_{1}{)}_{r-1}^{n} & \eta_{4}\\ 0 & \cdots & \cdots & \cdots & \cdots & 0 & \eta_{3} & (\eta_{1}{)}_{r}^{n} \end{array}),$$

and


$$𝒲=(\begin{array}{cccccccc} (\varrho_{1}{)}_{0}^{n} & \varrho_{2} & 0 & \cdots & \cdots & \cdots & \cdots & 0\\ \varrho_{3} & (\varrho_{1}{)}_{1}^{n} & \varrho_{4} & \ddots & & & & \vdots \\ 0 & \varrho_{3} & (\varrho_{1}{)}_{2}^{n} & \varrho_{4} & \ddots & & & \vdots \\ \vdots & \ddots & \ddots & \ddots & \ddots & \ddots & & \vdots \\ \vdots & & \ddots & \ddots & \ddots & \ddots & \ddots & \vdots \\ \vdots & & & \ddots & \varrho_{3} & (\varrho_{1}{)}_{r-2}^{n} & \varrho_{4} & 0\\ \vdots & & & & \ddots & \varrho_{3} & (\varrho_{1}{)}_{r-1}^{n} & \varrho_{4}\\ 0 & \cdots & \cdots & \cdots & \cdots & 0 & \varrho_{3} & (\varrho_{1}{)}_{r}^{n} \end{array}),$$

here,


$$(\chi_{1}{)}_{i}^{n}=1+\alpha_{2}+\rho (\frac{\beta_{1}I_{i}^{n}}{1+\alpha I_{i}^{n}})+2\varepsilon_{1},$$

$$(\nu_{1}{)}_{i}^{n}=1+(1+\alpha_{2}+\alpha_{4})\rho +2\varepsilon_{2},$$

$$(\eta_{1}{)}_{i}^{n}=1+2\varepsilon_{3}+\rho (\alpha_{2}+\alpha_{5}),$$

$$(\varrho_{1}{)}_{i}^{n}=1+\rho (\alpha_{1}+\alpha_{2})+2\varepsilon_{4},$$

$$\chi_{2}=-\varepsilon_{1},\;\;\nu_{2}=-2\varepsilon_{2},\;\;\eta_{2}=-2\varepsilon_{3},\;\;\varrho_{2}=-2\varepsilon_{4},$$

$$\chi_{3}=-\varepsilon_{1},\;\;\nu_{3}=-\varepsilon_{2},\;\;\eta_{3}=-\varepsilon_{3},\;\;\varrho_{3}=-\varepsilon_{4},$$

$$\chi_{4}=-\varepsilon_{1},\;\;\nu_{4}=-\varepsilon_{2},\;\;\eta_{4}=-\varepsilon_{3},\;\;\varrho_{4}=-\varepsilon_{4}.$$

The inductive technique id adopted to verify the positivity of the associated discrete system of equations (5)-(8). Initial conditions from (9)-(10) give ${\text{S}}^{0}$, ${\text{I}}^{0}$, ${\text{T}}^{0}$ and ${\text{R}}^{0}$ are positive also, suppose that ${\text{S}}^{\text{n}}$, ${\text{I}}^{\text{n}}$, ${\text{T}}^{\text{n}}$, and ${\text{R}}^{\text{n}}$, for n∈0,1,2,…,s−1, are positive component vectors. The above calculation indicates that 𝒯,𝒰,𝒱 and 𝒲 are the *M*-matrices, so, they are invertible and have positive inverses. Moreover, the expressions occurred on the right hand side of each of the equations in the system (24)-(27) is positive. Therefore,


$$S^{n+1}={𝒯}^{-1}(S_{i}^{n}+\lambda^{2}\rho (\Lambda +\alpha R_{i}^{n})),$$

$$I^{n+1}={𝒰}^{-1}(I_{i}^{n}+\rho (\frac{\beta_{1}I_{i}^{n}}{1+\alpha I_{i}^{n}}+\frac{\beta_{2}\alpha_{2}T_{i}^{n}}{1+\alpha T_{i}^{n}})S_{i}^{n}),$$

$$T^{k+1}={𝒱}^{-1}(T_{i}^{n}+P\alpha_{4}I_{i}^{n}),$$

$$R^{k+1}={𝒲}^{-1}(R_{i}^{n}+\rho (1-P)\alpha_{4}I_{i}^{n}+\alpha_{5}\rho T_{i}^{n}),$$

all the state variables are positive quantities for every n=0,1,2,···,s−1. Hence, the theory of mathematical induction grantees the required solutions. ◻

### 5.2 Consistency

In this subsection, an important structural property of proposed nonstandard finite difference scheme is analyzed. In this regard, an analytical result is established that verifies the consistency of numerical method [28]. It is important to memorize some definitions that can help to prove the result.

**Definition 5.1.** *Let $R_{\lambda }=\{x_{i}\in \mathbb{R}:i=1,2,\ldots ,r\}$ be a set of grid points and $V_{\lambda }$ be the set of real valued functions defined on $V_{\lambda }$. Also, let ‖.‖ and $\|.{\|}_{\infty }$ be the Euclidean and infinity norms on $V_{\lambda }$ respectively.*

Next, consider the differential operators E,F,G and H such that

$$E=S_{t}-\Lambda -\alpha_{1}R+\alpha_{2}S+(\frac{\beta_{1}I}{1+\alpha I}+\frac{\beta_{2}\alpha_{3}T}{1+\alpha T})S-d_{1}S_{xx},$$ (33)

$$F=I_{t}-(\frac{\beta_{1}I}{1+\alpha I}-\frac{\beta_{2}\alpha_{3}T}{1+\alpha T})S+(\alpha_{2}+\alpha_{4})I-d_{2}I_{xx},$$ (34)

$$G=T_{t}-P\alpha_{4}I+(\alpha_{2}+\alpha_{5})T-d_{3}T_{xx},$$ (35)

$$H=R_{t}-(1-P)\alpha_{4}I-\alpha_{5}T+(\alpha_{1}+\alpha_{2})R-d_{4}R_{xx},$$ (36)

It is important to note that $E_{i}^{n}=E(x_{i},t_{n})$, $F_{i}^{n}=F(x_{i},t_{n})$, $G_{i}^{n}=G(x_{i},t_{n})$ and $H_{i}^{n}=H(x_{i},t_{n})$ where i∈{0,1,2,···,r} and n∈{0,1,2,···,s}.

For each n∈{0,1,2,···,s}, we can define $E^{n}=(E_{0}^{q},E_{1}^{q},\ldots ,E_{r}^{q})$, $F^{n}=(F_{0}^{q},F_{1}^{q},\ldots ,F_{r}^{q})$, $G^{n}=(G_{0}^{q},G_{1}^{q},\ldots ,G_{r}^{q})$, $H^{n}=(H_{0}^{q},H_{1}^{q},\ldots ,H_{r}^{q})$.

This shows that ${\text{E}}^{\text{n}}$, ${\text{F}}^{\text{n}}$, ${\text{G}}^{\text{n}}$, $H^{n}\in V_{\lambda }$ and finally, we have *E* = (*E*^*n*^), *F* = (*F*^*n*^), *G* = (*G*^*n*^) and *H* = (*H*^*n*^), for n∈{0,1,2,···,s}.

Now, in the same way, we can make the assumptions for the discrete system (20)-(23).

$$𝔈=\delta_{t}S_{i}^{n+1}-\Lambda -\alpha_{1}R_{i}^{n}+\alpha_{2}S_{i}^{n+1}+(\frac{\beta_{1}I_{i}^{n}}{1+\alpha I_{i}^{n}}+\frac{\beta_{2}\alpha_{3}T_{i}^{n}}{1+\alpha T_{i}^{n}})S_{i}^{n+1}-\;d_{1}\delta_{xx}S_{i}^{n+1},$$ (37)

$$𝔉=\delta_{t}I_{i}^{n+1}-(\frac{\beta_{1}I_{i}^{n}}{1+\alpha I_{i}^{n}}+\frac{\beta_{2}\alpha_{3}T_{i}^{n}}{1+\alpha T_{i}^{n}})S_{i}^{n}+(\alpha_{2}+\alpha_{4})I_{i}^{n+1}-\;d_{2}\delta_{xx}I_{i}^{n+1},$$ (38)

$$𝔊=\delta_{t}T_{i}^{n+1}-P\alpha_{4}I_{i}^{n}+(\alpha_{2}+\alpha_{5})T_{i}^{n+1}-d_{3}\delta_{xx}T_{i}^{n+1},$$ (39)

$$ℌ=\delta_{t}R_{i}^{n+1}-(1-P)\alpha_{4}I_{i}^{n}-\alpha_{5}T_{i}^{n}+(\alpha_{1}+\alpha_{2})R_{i}^{n+1}-d_{4}\delta_{xx}R_{i}^{xx}.$$ (40)

**Theorem 5.2. (Consistency)** *Assume that S, I, T and R be the functions having continuous second order partial derivatives with in the domain, then for a constant 𝕂, independent of r and s such that*

$$max\{\|E-𝔈{\|}_{\infty },\|F-𝔉{\|}_{\infty },\|G-𝔊{\|}_{\infty },\|H-ℌ{\|}_{\infty }\}\leq s𝕂.$$ (41)

*Proof:* Since *S* is continuous and bounded on the domain ℱ and Taylor’s theorem is applicable on *S* then there exist some nonnegative constant numbers ${𝕂}_{1}^{S},{𝕂}_{2}^{S},{𝕂}_{3}^{S}$ and ${𝕂}_{4}^{S}$ with the property that these numbers are independent of *r* and *s* such that *S* satisfies the following inequalities:

$$|\frac{\partial S(x_{i},t_{n+1})}{\partial t}-\delta_{t}S_{i}^{n+1}|\leq \rho {𝕂}_{1}^{S},$$ (42)

$$|R(x_{i},t_{n+1})-R_{i}^{n}|\leq \rho {𝕂}_{2}^{S},$$ (43)

$$|\frac{\partial^{2}S(x_{i},t_{n+1})}{\partial x^{2}}-\delta_{xx}S_{i}^{n+1}|\leq \lambda {𝕂}_{3}^{S}.\;$$ (44)

Now, (42),(43) and (44) can be unite to get

$$\|E-𝔈{\|}_{\infty }\leq (\rho +\lambda ){𝕂}^{S}.$$ (45)

The inequality (45) holds if the constant ${𝕂}^{S}={𝕂}_{1}^{S}+\omega_{1}{𝕂}_{2}^{S}+\omega_{2}{𝕂}_{3}^{S}$ is defined. Similarly, we can make the inequalities $\|F-𝔉{\|}_{\infty }\leq (\rho +\lambda ){𝕂}^{I}$, $\|G-𝔊{\|}_{\infty }\leq (\rho +\lambda ){𝕂}^{T}$, and $\|H-ℌ{\|}_{\infty }\leq (\rho +\lambda ){𝕂}^{R}$, where the constants ${𝕂}^{S}$, ${𝕂}^{I}$, ${𝕂}^{T}$, and ${𝕂}^{R}$ are independent of ρ and *lambda*. The result of the current theorem can be drawn if we define a constant $𝕂={𝕂}^{S}$ ∨ ${𝕂}^{I}$ ∨ ${𝕂}^{T}$ ∨ ${𝕂}^{R}$ which is also independent of ρ and λ. ◻

**Theorem 5.3. (Linear Stability)** *If the functions* $S_{0}(x),I_{0}(x),T_{0}(x)$, and *R*_*0*_*(*x*) are positive for all $x\in \bar{\Omega }$ then the discrete system (*20*)-(*23*) is stable in Von Neumann sense.*

*Proof:* The proof of the theorem is straight forward. ◻

Before establishing the theorem about nonlinearity of the discrete model (20)-(23), it is important to recall the Gronwall’s inequality in discrete form.

**Lemma 5.1.** *[29] Suppose that for a nonnegative number θ, $(\zeta^{n}{)}_{n\in \{0,1,2,\cdot \cdot \cdot ,s\}}$ and $(\varsigma^{n}{)}_{n\in \{0,1,2,\cdot \cdot \cdot ,s\}}$ be the finite sequences of nonnegative real numbers. Also, let, for a nonnegative constant number 𝒞, the inequality*

$$\zeta^{j+1}\leq \varsigma^{j+1}+\theta 𝒞\sum_{n=0}^{j}\zeta^{n},\text{ for }j\in \{0,1,2,\cdot \cdot \cdot ,s-1\},$$ (46)

*holds. Then $\zeta^{n}\leq \varsigma^{n}e^{n𝒞\theta }$, ∈{0,1,2,···,s}.*

Before starting the next theorem, we make some assumptions that will help to establish the result.

Define the functions $ℑ=\frac{I_{i}^{n}}{1+\alpha I_{i}^{n}}$ and $𝔗=\frac{T_{i}^{n}}{1+\alpha T_{i}^{n}}$ with ${\upsilon^{*}}_{i}^{n}={ℑ}_{i}^{n}-{\tilde{ℑ}}_{i}^{n}$ and ${\tau^{*}}_{i}^{n}={𝔗}_{i}^{n}-{\tilde{𝔗}}_{i}^{n}$ such that these functions together with the numerical solutions are uniformly bounded so, let ${𝔅}_{0}$ be their common bound at a point $\left(x_{i},t_{n}\right)$, for i∈{0,1,2,···,r} and n∈{0,1,2,···,s}.

Also, we can define a number $C^{\prime }=2\alpha_{1}+4\alpha_{2}+2\alpha_{4}+2\alpha_{5}+4\beta_{1}{𝔅}_{0}+4\alpha_{3}\beta_{2}{𝔅}_{0}+\frac{16}{h}(d_{1}+d_{2}+d_{3}+d_{4})$ such that $\rho C^{\prime }<1$.

**Theorem 5.4. (Nonlinear Stability)** *Suppose that $\left(S_{0},I_{0},T_{0},R_{0}\right)$ and $\left(\tilde{S_{0}},\tilde{I_{0}},\tilde{T_{0}},\tilde{R_{0}}\right)$ be the set of positive functions representing the initial conditions for the system (*5*)-(*8*). Also, let (S, I, T, R) and $\left(\tilde{S},\tilde{I},\tilde{T},\tilde{R}\right)$ be the respected sets of bounded solution functions to the system (*20*)-(*23*) with the conditions (*28*). Define*

$$\omega^{k}=\|\epsilon^{k}\|+\|\upsilon^{k}\|+\|\tau^{k}\|+\|\vartheta^{k}\|,\;\text{ for all }k\in \{0,1,2,\cdot \cdot \cdot s\}.$$ (47)

$$\text{ and }\varsigma =(\|\epsilon^{0}\|+\|\upsilon^{0}\|+\|\tau^{0}\|+\|\vartheta^{0}\|)(1-\rho C^{\prime }{)}^{-1}.$$ (48)

*Then there exist a nonnegative constant C, depending upon both ρ and λ such that*


$$\omega^{n}\leq \varsigma e^{nC\rho },\text{ for each }n\in \{0,1,2,\cdot \cdot \cdot ,s\}.$$

*Proof:* The system satisfied by the point $\left(\tilde{S_{0}},\tilde{I_{0}},\tilde{T_{0}},\tilde{R_{0}}\right)$ can be written according to (20)-(23) is

$$\delta_{t}{\tilde{S}}_{i}^{n+1}=\Lambda +\alpha_{1}{\tilde{R}}_{i}^{n}-\alpha_{2}{\tilde{S}}_{i}^{n+1}-(\frac{\beta_{1}{\tilde{I}}_{i}^{n}}{1+\alpha {\tilde{I}}_{i}^{n}}+\frac{\beta_{2}\alpha_{3}{\tilde{T}}_{i}^{n}}{1+\alpha {\tilde{T}}_{i}^{n}}){\tilde{S}}_{i}^{n+1}+\;d_{1}\delta_{xx}{\tilde{S}}_{i}^{n+1},$$ (49)

$$\delta_{t}{\tilde{I}}_{i}^{n+1}=(\frac{\beta_{1}{\tilde{I}}_{i}^{n}}{1+\alpha {\tilde{I}}_{i}^{n}}+\frac{\beta_{2}\alpha_{3}{\tilde{T}}_{i}^{n}}{1+\alpha {\tilde{T}}_{i}^{n}}){\tilde{S}}_{i}^{n}-(\alpha_{2}+\alpha_{4}){\tilde{I}}_{i}^{n+1}+d_{2}\delta_{xx}{\tilde{I}}_{i}^{n+1},$$ (50)

$$\delta_{t}{\tilde{T}}_{i}^{n+1}=P\alpha_{4}{\tilde{I}}_{i}^{n}-(\alpha_{2}+\alpha_{5}){\tilde{T}}_{i}^{n+1}+d_{3}\delta_{xx}{\tilde{T}}_{i}^{n+1},$$ (51)

$$\delta_{t}{\tilde{R}}_{i}^{n+1}=(1-P)\alpha_{4}{\tilde{I}}_{i}^{n}+\alpha_{5}{\tilde{T}}_{i}^{n}-(\alpha_{1}+\alpha_{2}){\tilde{R}}_{i}^{n+1}+d_{4}\delta_{xx}{\tilde{R}}_{i}^{n+1}.$$ (52)

The corresponding discrete condition for the system (49)-(52),

$${\tilde{S}}_{i}^{0}={\tilde{S}}_{0}(x_{i}),\;\;\text{ for all }i\in \{0,1,2,\cdot \cdot \cdot ,r-1\},\;{\tilde{I}}_{i}^{\;0}={\tilde{I}}_{0}(x_{i}),\;\;\;\text{ for all }i\in \{0,1,2,\cdot \cdot \cdot ,r-1\},\;{\tilde{T}}_{i}^{\;0}={\tilde{T}}_{0}(x_{i}),\;\text{ for all }i\in \{0,1,2,\cdot \cdot \cdot ,r-1\},\;{\tilde{R}}_{i}^{0}={\tilde{R}}_{0}(x_{i}),\;\text{ for all }i\in \{0,1,2,\cdot \cdot \cdot ,r-1\},\;\text{and}\;\delta_{x}{\tilde{S}}_{1}^{n}=\delta_{x}{\tilde{I}}_{1}^{n}=\delta_{x}{\tilde{T}}_{1}^{\;n}=\delta_{x}{\tilde{R}}_{1}^{n}=0,\;\text{ for all }n\in \{0,1,2,\cdot \cdot \cdot ,s\},\;\delta_{x}{\tilde{S}}_{r}^{n}=\delta_{x}{\tilde{I}}_{r}^{n}=\delta_{x}{\tilde{T}}_{r}^{n}=\delta_{x}{\tilde{R}}_{r}^{n}=0,\;\text{ for all }n\in \{0,1,2,\cdot \cdot \cdot ,s\}.$$ (53)

Now, we can define a 4-tuple (ϵ,υ,τ,ϑ) such as

$$\epsilon_{i}^{0}=S_{0}(x_{i})-{\tilde{S}}_{0}(x_{i}),\;\upsilon_{i}^{0}=I_{0}(x_{i})-{\tilde{I}}_{0}(x_{i}),\;\tau_{i}^{0}=T_{0}(x_{i})-{\tilde{T}}_{0}(x_{i}),\;\vartheta_{i}^{0}=R_{0}(x_{i})-{\tilde{R}}_{0}(x_{i}),\;\text{ for all }i\in \{0,1,2,\cdot \cdot \cdot ,r\}\text{ and }n\in \{0,1,2,\cdot \cdot \cdot ,s\}.$$ (54)

It is noticed that the point (ϵ,υ,τ,ϑ) also satisfies the difference equations

$$\delta_{t}\epsilon_{i}^{n+1}=\alpha_{1}\vartheta_{i}^{n}-\alpha_{2}\epsilon_{i}^{n+1}-(\frac{\beta_{1}I_{i}^{n}}{1+\alpha I_{i}^{n}}+\frac{\beta_{2}\alpha_{3}T_{i}^{n}}{1+\alpha T_{i}^{n}})S_{i}^{n+1}+\;(\frac{\beta_{1}{\tilde{I}}_{i}^{n}}{1+\alpha {\tilde{I}}_{i}^{n}}+\frac{\beta_{2}\alpha_{3}{\tilde{T}}_{i}^{n}}{1+\alpha {\tilde{T}}_{i}^{n}}){\tilde{S}}_{i}^{n+1}+d_{1}\delta_{xx}\epsilon_{i}^{n+1},$$ (55)

$$\delta_{t}\upsilon_{i}^{n+1}=(\frac{\beta_{1}I_{i}^{n}}{1+\alpha I_{i}^{n}}+\frac{\beta_{2}\alpha_{3}T_{i}^{n}}{1+\alpha T_{i}^{n}})S_{i}^{n+1}-(\frac{\beta_{1}{\tilde{I}}_{i}^{n}}{1+\alpha {\tilde{I}}_{i}^{n}}+\frac{\beta_{2}\alpha_{3}{\tilde{T}}_{i}^{n}}{1+\alpha {\tilde{T}}_{i}^{n}}){\tilde{S}}_{i}^{n+1}-\;(\alpha_{2}+\alpha_{4})\upsilon_{i}^{n+1}+d_{2}\delta_{xx}\upsilon_{i}^{n+1},$$ (56)

$$\delta_{t}\tau_{i}^{n+1}=P\alpha_{4}\upsilon_{i}^{n}-(\alpha_{2}+\alpha_{5})\tau_{i}^{n+1}+d_{3}\delta_{xx}\tau_{i}^{n+1},$$ (57)

$$\delta_{t}\vartheta_{i}^{n+1}=(1-P)\alpha_{4}\upsilon_{i}^{n}+\alpha_{5}\tau_{i}^{n}-(\alpha_{1}+\alpha_{2})\vartheta_{i}^{n+1}+d_{4}\delta_{xx}\vartheta_{i}^{n+1},$$ (58)

with the initial and boundary conditions

$$S_{i}^{0}=S_{0}(x_{i}),\;I_{i}^{\;0}=I_{0}(x_{i}),\;T_{i}^{\;0}=T_{0}(x_{i}),\;R_{i}^{0}=R_{0}(x_{i}),\;\text{for}\;i\in \{1,2,\cdot \cdot \cdot ,r\},\;\text{ and }\;\delta S_{1}^{n}=\delta I_{1}^{n}=\delta T_{1}^{\;n}=\delta R_{1}^{n}=0,\;\delta S_{r}^{n}=\delta I_{r}^{n}=\delta T_{r}^{n}=\delta R_{r}^{n}=0,\;\text{for}\;n\in \{1,2,\cdot \cdot \cdot ,s\}.$$ (59)

Rearranging the terms of [a42]55 and applying the formulas of discretization, we get

$$\epsilon_{i}^{n+1}-\epsilon_{i}^{n}=\varepsilon_{1}(\epsilon_{i+1}^{n+1}-2\epsilon_{i}^{n+1}+\epsilon_{i-1}^{n+1})+\rho \alpha_{1}\vartheta_{i}^{n}-\rho \alpha_{2}\epsilon_{i}^{n+1}-\;\rho \beta_{1}\{{ℑ}_{i}^{n}S_{i}^{n+1}-{\tilde{ℑ}}_{i}^{n}{\tilde{S}}_{i}^{n_{1}}\}-\rho \beta_{2}\alpha +2\{\{{𝔗}_{i}^{n}S_{i}^{n+1}-{\tilde{𝔗}}_{i}^{n}{\tilde{S}}_{i}^{n_{1}}\},$$ (60)

for each i∈{1,2,···,r} and n∈{0,1,2,···,s} and $\varepsilon_{1}=\frac{\rho d_{1}}{\lambda^{2}}$.

Now, by applying the Euclidean norm on both sides and using the properties of the norm, we have


$$\|\epsilon^{n+1}\|-\|\epsilon^{n}\|\leq 4\varepsilon_{1}\|\epsilon^{n+1}\|+\rho \alpha_{1}\|\vartheta^{n}\|+\rho \alpha_{2}\|\epsilon^{n+1}\|+\rho \beta_{1}\|S^{n+1}{\upsilon^{*}}^{n}\|+\;\rho \beta_{1}\|\epsilon^{n+1}{\tilde{𝔗}}^{n}\|+\rho \alpha_{3}\beta_{2}\|S^{n+1}{\tau^{*}}^{n}\|+\rho \beta_{2}\alpha_{3}\|\epsilon^{n+1}{\tilde{𝔗}}^{n}\|,$$

$$\|\epsilon^{n+1}\|-\|\epsilon^{n}\|\leq 4\varepsilon_{1}\|\epsilon^{n+1}\|+\rho \alpha_{1}\|\vartheta^{n}\|+\rho \alpha_{2}\|\epsilon^{n+1}\|+\rho \beta_{1}{𝔅}_{0}\|{\upsilon^{*}}^{n}\|+\;\rho \beta_{1}{𝔅}_{0}\|\epsilon^{n+1}\|+\rho \alpha_{3}\beta_{2}{𝔅}_{0}\|{\tau^{*}}^{n}\|+\rho \alpha_{3}\beta_{2}{𝔅}_{0}\|\epsilon^{n+1}\|,$$

$$\|\epsilon^{n+1}\|-\|\epsilon^{n}\|\leq (4\varepsilon_{1}+\rho \alpha_{2}+\rho \beta_{1}{𝔅}_{0}+\rho \alpha_{3}\beta_{2}{𝔅}_{0})\|\epsilon^{n+1}\|+\rho \alpha_{1}\|\vartheta^{n}\|+\;\rho \beta_{1}{𝔅}_{0}\|{\upsilon *}^{n}\|+\rho \alpha_{3}\beta_{2}{𝔅}_{0}\|{\tau^{*}}^{n}\|,$$

for each n∈{1,2,···,s−1}.

Let

$$C_{1}=4\frac{d_{1}}{\lambda^{2}}+\alpha_{2}+\beta_{1}{𝔅}_{0}\alpha_{3}\beta_{2}{𝔅}_{0}.$$ (61)

Then


$$\|\epsilon^{n+1}\|-\|\epsilon^{n}\|\leq \rho C_{1}\|\epsilon^{n+1}\|+\rho \alpha_{1}\|\vartheta^{n}\|+\rho \beta_{1}{𝔅}_{0}\|{\upsilon^{*}}^{n}\|+\rho \alpha_{3}\beta_{2}+\;\rho \beta_{1}{𝔅}_{0}\|{\tau^{*}}^{n}\|,$$

Now, let k∈{1,2,···,s} and taking summation on both sides for n∈{0,1,2,···,k}. Also, using telescopic sum on the left hand side, we get

$$\|\epsilon^{k+1}\|\leq \|\epsilon^{0}\|+C_{1}^{\prime }\rho \sum_{n=0}^{k+1}\omega^{n},\text{ for all }k\in \{1,2,\cdot \cdot \cdot ,s-1\},$$ (62)

where


$$C_{1}^{\prime }=C_{1}+\alpha_{1}+\beta_{1}{𝔅}_{0}+\alpha_{3}\beta_{2}{𝔅}_{0},$$

$$=\frac{4d_{1}}{\lambda^{2}}+\alpha_{2}+\beta_{1}{𝔅}_{0}+\beta_{2}{𝔅}_{0}+\alpha_{1}+\beta_{1}{𝔅}_{0}+\alpha_{3}\beta_{2}{𝔅}_{0},$$

$$=\frac{4d_{1}}{\lambda^{2}}+\alpha_{1}+\alpha_{2}+2\beta_{1}{𝔅}_{0}+2\alpha_{3}\beta_{2}{𝔅}_{0}.$$ (63)

Again, rearranging the terms of [a43]56, [a44]57 and [a45]58 and applying the formulas of discretization, we have

$$\upsilon_{i}^{n+1}-\upsilon_{i}^{n}=\rho \beta_{1}{ℑ}_{i}^{n}S_{i}^{n+1}-\rho \beta_{1}{\tilde{ℑ}}_{i}^{n}{\tilde{S}}_{i}^{n+1}+\rho \beta_{2}\alpha_{3}{𝔗}_{i}^{n}S_{i}^{n+1}-\rho \beta_{2}\alpha_{3}{\tilde{T}}_{i}^{n}{\tilde{S}}_{i}^{n+1}-\;\rho (\alpha_{2}+\alpha_{4})\upsilon_{i}^{n+1}+\varepsilon_{2}(\upsilon_{i+1}^{n+1}-2\upsilon_{i}^{n+1}+\upsilon_{i-1}^{n+1}).$$ (64)

$$\tau_{i}^{n+1}-\tau_{i}^{n}=\rho P\alpha_{4}\upsilon_{i}^{n}-\rho (\alpha_{2}+\alpha_{5})\tau_{i}^{n+1}+\varepsilon_{3}(\tau_{i+1}^{n+1}-2\tau_{i}^{n+1}+\tau_{i-1}^{n+1}),$$ (65)

$$\vartheta_{i}^{n+1}-\vartheta_{i}^{n}=\rho (1-P)\alpha_{4}\upsilon_{i}^{n}+\rho \alpha_{5}\tau_{i}^{n}-\rho (\alpha_{1}+\alpha_{2})\vartheta_{i}^{n+1}+\;\varepsilon_{4}(\vartheta_{i+1}^{n+1}-2\vartheta_{i}^{n+1}+\vartheta_{i-1}^{n+1}),$$ (66)

where

$$\varepsilon_{2}=\frac{\rho d_{2}}{\lambda^{2}},\;\varepsilon_{3}=\frac{\rho d_{3}}{\lambda^{3}},\;\varepsilon_{4}=\frac{\rho d_{4}}{\lambda^{4}}.$$ (67)

After applying Euclidean norm on (56), (57) and (58), we get

$$\|\upsilon^{k+1}\|\leq \|\upsilon^{0}\|+C_{2}^{\prime }\rho \sum_{n=0}^{k+1}\omega^{n},\text{ for all }k\in \{1,2,\cdot \cdot \cdot ,s-1\},$$ (68)

$$\|\tau^{k+1}\|\leq \|\tau^{0}\|+C_{3}^{\prime }\rho \sum_{n=0}^{k+1}\omega^{n},\text{ for all }k\in \{1,2,\cdot \cdot \cdot ,s-1\},$$ (69)

$$\|\vartheta^{k+1}\|\leq \|\vartheta^{0}\|+C_{4}^{\prime }\rho \sum_{n=0}^{k+1}\omega^{n},\text{ for all }k\in \{1,2,\cdot \cdot \cdot ,s-1\},$$ (70)

in which (68), (69) and (68), $C_{2}^{\prime },C_{3}^{\prime }$ and $C_{4}^{\prime }$ are

$$C_{2}^{\prime }=\frac{4d_{2}}{\lambda^{2}}+\alpha_{2}+\alpha_{4}+2\beta_{1}{𝔅}_{0}+2\alpha_{3}\beta_{2}{𝔅}_{0},$$ (71)

$$C_{3}^{\prime }=\frac{4d_{3}}{\lambda^{2}}+\alpha_{2}+\alpha_{5}+P\alpha_{4},$$ (72)

$$C_{4}^{\prime }=\frac{4d_{4}}{\lambda^{2}}+\alpha_{1}+\alpha_{2}+(1-P)\alpha_{4}+\alpha_{5}.$$ (73)

Adding (62), (68), (69) and (70)


$$\|\epsilon^{k+1}\|+\|\upsilon^{k+1}\|+\|\tau^{k+1}\|+\|\vartheta^{k+1}\|\leq \|\epsilon^{0}\|+\|\upsilon^{0}\|+\|\tau^{0}\|+\|\vartheta^{0}\|+$$

$$C_{1}^{\prime }\rho \sum_{n=0}^{k+1}\omega^{n}+C_{2}^{\prime }\rho \sum_{n=0}^{k+1}\omega^{n}+C_{3}^{\prime }\rho \sum_{n=0}^{k+1}\omega^{n}+C_{3}^{\prime }\rho \sum_{n=0}^{k+1}\omega^{n}.$$

This gives


$$\omega^{k+1}\leq \omega^{0}+\rho C^{\prime }\sum_{n=0}^{k+1}\omega^{n},$$

$$\omega^{k+1}\leq \omega^{0}+\rho C^{\prime }\sum_{n=0}^{k}\omega^{n}+\rho C^{\prime }\omega^{n+1},$$

$$(1-\rho C^{\prime })\omega^{k+1}\leq \omega^{0}+\rho C^{\prime }\sum_{n=0}^{k}\omega^{n},$$

$$\omega^{k+1}\leq \frac{\omega^{0}}{1-\rho C^{\prime }}+\frac{\rho C^{\prime }}{1-\rho C^{\prime }}\sum_{n=0}^{k}\omega^{n},$$

It can be written in the form

$$\omega^{k+1}\leq \varsigma +C\sum_{n=0}^{k}\omega^{n},\text{ where }\varsigma =\frac{\omega^{0}}{1-\rho C^{\prime }}\text{ and }C=\frac{C^{\prime }}{1-\rho C^{\prime }}.$$ (74)

Also, $\omega^{0}=\|\epsilon^{0}\|+\|\upsilon^{0}\|+\|\tau^{0}\|+\|\vartheta^{0}\|$ and $\varsigma^{n}=\varsigma$. It is clear from (73) that all the conditions of Lemma 11 are satisfied, so the conclusion of the theorem is followed from the Lemma 11. ◻

## 6 Results and discussions

**Fig 1:**
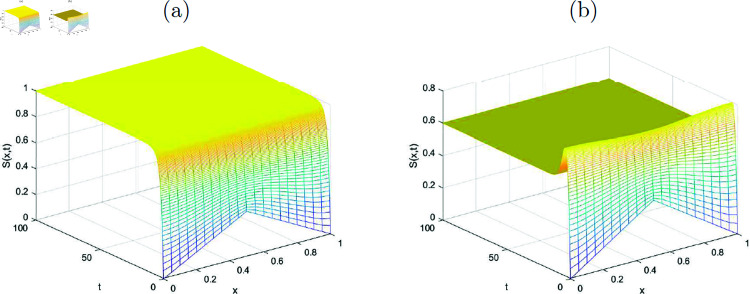
(a) Disease-free state, (b) Endemic state representing numerical approximations for the function S(x, t) (susceptible individuals) with k=10.3.

**Fig 2:**
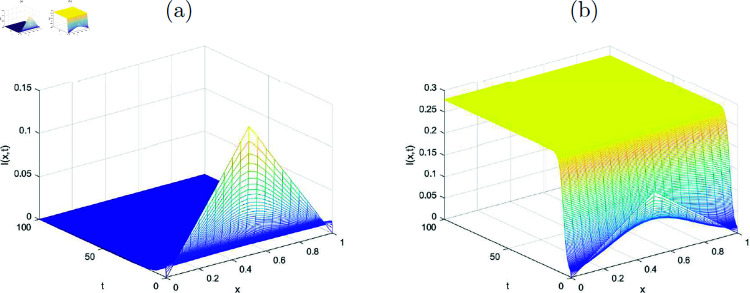
(a) Disease-free state, (b) Endemic state representing numerical approximations for the function I(x, t) (infected class) with k=10.3.

**Fig 3:**
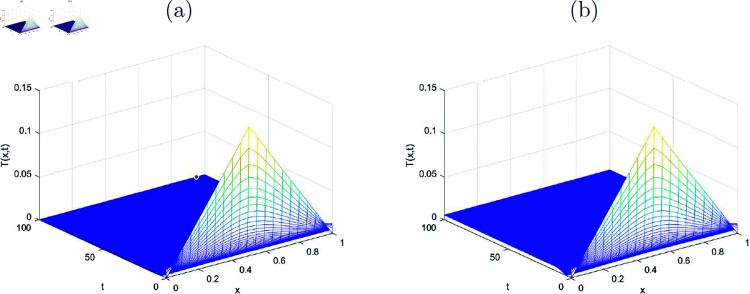
(a) Disease-free state, (b) Endemic state representing numerical approximations for the function T(x, t) (treated class) with k=10.3.

**Fig 4:**
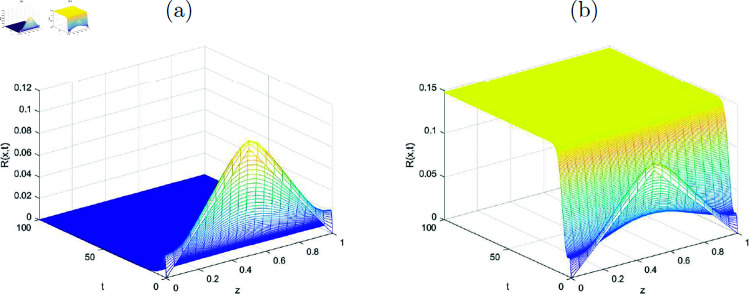
(a) Disease-free state, (b) Endemic state, representing numerical approximations for the function R(x, t) (recovered class) with k=10.3.

In this section, the simulated graphs of the discretized state variables of the system (24)-(27) are presented. Each figure comprised of two sub figures (*a*) and (*b*) where (*a*) in each figure, represent the graphs of disease-free equilibrium and (*b*) represent the endemic equilibrium states of the state variables in each figure. The graphs of the state variables at disease free equilibrium pointy *E*_*df*_ are plotted where the value of *R*_0_<1 (evaluated by using the values of the parameters given in Table 1) for each of the graphs. The Figure 1(a) shows the convergence of the susceptible individuals towards the disease free equilibrium point for various values of *x* and *t*. Similarly, the Figures 2(a), 3(a) and 4(a) reflect the convergence of the implicit numerical scheme towards the true steady-state, which is zero for every state variable, i.e., for *I*(*x*, *t*), *T*(*x*, *t*) and *R*(*x*, *t*). Likewise, the behavoiur of the state variables at endemic equilibrium point is drawn in the upcoming figures. Stability of the endemic equilibrium state can be observed by taking those parameters that give *R*_0_>1, as given in the Table 1. The spatio-temporal graphs in Figures 1(a)-4(a) reveal the nonlinear behavior and convergence towards the exact fixed point which is endemic equilibrium point *E*_*e*_ in this case for *R*_0_>1.

## 7 Conclusion

The current paper has quantitatively and numerically investigated a nonlinear reaction-diffusion epidemic model of diarrhea disease. The model is made more realistic as well as exclusive by incorporating the spatial diffusion. It gives more information about the dynamics of disease across space. By vanishing all the instantaneous rates of changes, equilibrium points for the model and the basic reproduction number *R*_0_ are calculated. The positivity and boundedness which play a crucial role in analyzing the continuous population model are also verified by some analytical techniques. Global stability of the steady states of the system with diffusion is justified with the help of the Lyapunov function. To obtain the approximate solution of the prescribed model, a nonstandard finite difference method (NSFD) is applied to the underlying system and it is proved that the NSFD is consistent with the system under study. For the reliability of the numerical scheme, it is applied to a the proposed system. It is necessary to preserve all the physical features of the continuous model by the numerical scheme. In this respect, the positivity, boundedness, and nonlinear stability results are developed. The consistency, stability, and positivity of the nonstandard finite difference scheme are also proved for the current system. In the end, numerical simulations are drawn to validate the theoretical results.
